# Supplementary material for: SyNDI: synchronous network data integration framework
Source: BMC Bioinformatics. 2018 Nov 6;19:403. doi: 10.1186/s12859-018-2426-5 (PMC6219086; doi:10.1186/s12859-018-2426-5)
Supplement: Supplementary file 5 — Files of the example presented in Additional file 4. This additional file is a zip package that contains all files used in Additional file 4. Detailed descriptions of each of these files are presented in a README file included in this zip package. (GZ 30411 kb) [file 12859_2018_2426_MOESM5_ESM.gz › SyNDITools/Meme2Fimo/meme_4.9.0_4.tar.gz!/meme_4.9.0/doc/mhmm.xml]

mhmm


# mhmm -- not currently supported

**Usage:** mhmm [options] <*MEME file*>

**Description:**

This program creates motif-based hidden Markov models
(HMMs) of families of related biosequences. The program takes as
input a set of DNA or protein motif models constructed by MEME and produces as output a single
HMM containing the given motifs. `mhmm` can produce
three types of models: linear models, in which the motifs are
arranged like beads on a string, completely connected models, which
allow for repetitions of motifs and for motifs to appear in any
order, and star models. `mhmm` writes its output in a
format readable by the other Meta-MEME programs, mhmms and mhmmscan.

Three types of models may be produced. A linear motif-based HMM
consists of a sequence of motif models, each separated by one or
more tied insert states that represent the spacer region between
motifs. A completely connected model, on the other hand, includes
transitions from the end of each motif to the beginning of every
other motif in the model (with a spacer model along each
transition). This more general topology allows for motifs that are
repeated, deleted or shuffled. A star model is also available. By
default, the program produces a linear model.

Transition probabilities among motifs are derived from the motif
occurence information in the given MEME file. For the completely
connected topology, this information is derived from all of the
motif occurences. For the linear topology, the information is
derived only from the best-scoring sequence. Alternatively, the
order and spacing of motifs within a linear model may be specified
via the "--order" option.

**Input:**

- <*MEME file*> - a file containing one or more motifs
  in MEME format.

**Output:**

**The Meta-MEME model file contains the following
information:**

- The Meta-MEME version number and release date.
- The program name, file contents and date the file was
  created.
- A link to the Meta-MEME home page.
- A reference to cite if you publish results from Meta-MEME.
- Some global information about the Meta-MEME model.
- A list of the states in the Meta-MEME model.
- The matrix of transition probabilities between states of the
  model.

**HMM Global Information**

The list of states in the Meta-MEME model begins with eight lines
that specify the global characteristics of the model. These lines
tell

- whether the model has a linear or completely connected topology
  (i.e., whether the model allows for repetition and shuffling of
  motifs),
- the total number of states in the model,
- the total number of spacers (i.e., inter-motif regions) in the
  model,
- the number of states used to represent each spacer,
- the number of letters in the alphabet (4 for DNA and 20 for
  amino acids),
- the alphabet,
- the background frequencies used as emission probabilities in
  the spacer states, and
- the name of the MEME output file that was used to generate this
  Meta-MEME model.

**HMM States**

Following the global characteristics is a list of state
descriptions. Each state description consists of six lines. These
lines tell

- the index of the state (numbered from 0),
- the type of state (i.e, whether it is a start or end state, a
  spacer state, or the beginning, middle or end of a motif),
- the index of the motif within which this state appears (This
  value is arbitrarily set to -1 for non-motif states.),
- the position of the state within the motif or spacer region,
  and
- the letter that is printed above this state when the MEME motif
  ID is printed centered with respect to the motif,
- the list of probabilities that this state will emit each of the
  letters in the alphabet.

The final item (the list of emission probabilities) is omitted
from the start and end states, which do not emit letters.

**Transition probability matrix**

The Meta-MEME model file ends with a square matrix containing n
rows and n columns, where n is the number of states in the model.
The entry in row i, column j of this matrix is the probability of
transitioning from state i to state j in the model. Consequently,
each row in the matrix sums to 1.0.

**Options:**

The program allows five different ways of selecting motifs from
the input file. The default method is to select all the motifs. The
first four command-line options provide alternative means of
selecting motifs. All of these four options are mutually
exclusive.

- --motif <*motif #*> - This option (which may be
  repeated) allows the user to select a specific motif for inclusion
  in the HMM. The specified motif number corresponds to the motif
  index in the MEME file.
- --nmotifs <*n*> - This option is similar to the
  '-motif' option, except that it tells `mhmm` to use the
  first n motifs in the given MEME file.
- --ethresh <*E-value*> - This option sets an E-value
  threshold for inclusion of motifs in the model. Motifs with
  E-values below the specified value are ignored.
- --lowcomp <*threshold*> - Eliminate low-complexity
  motifs from the model. Motif complexity is the average K-L distance
  between the "motif background distribution" and each column of the
  motif. The motif background is just the average distribution of all
  the columns. The K-L distance, which measures the difference
  between two distributions, p and f, is the same as the information
  content:
  p_1_ log(p_1_/f_1_) +
  p_2_ log(p_2_/f_2_) + ... +
  p_n_ log(p_n_/f_n_).
  This value increases with increasing complexity.
- --order <*string*> - This option instructs
  `mhmm` to build an HMM with linear topology. The given
  string specifies the order and spacing of the motifs within the
  model, and has the format "l=n=l=n=...=l=n=l", where "l" is the
  length of a region between motifs, and "n" is a motif index. Thus,
  for example, the string "34=3=17=2=5" specifies a two-motif linear
  model, with motifs 3 and 2 separated by 17 letters and flanked by
  34 letters and 5 letters on the left and right. If the MEME file
  contains motif occurrences on both strands, then the motif IDs in
  the order string should be preceded by "+" or "-" indicating the
  strandedness of the motif.
- --type [linear|complete|star] - A linear motif-based HMM
  consists of a sequence of motif models, each separated by one or
  more tied insert states that represent the spacer region between
  motifs. A completely connected model, on the other hand, includes
  transitions from the end of each motif to the beginning of every
  other motif in the model (with a spacer model along each
  transition). This more general topology allows for motifs that are
  repeated, deleted or shuffled. By default, the program produces a
  linear model. The order and spacing of motifs in the model is
  determined by the single sequence with the lowest E-value.
- --pthresh <*p-value*> - This option sets a p-value
  threshold for inclusion of motif occurences in the transition
  probability matrix used to construct the HMM.
- --nspacer <*value*> - By default, `mhmm`
  models each spacer using a single insert state. The distribution of
  spacer lengths produced by a single insert state is exponential in
  form. A more reasonable distribution would be a bell-shaped curve
  such as a Gaussian. Modeling the length distribution explicitly is
  computationally expensive; however, a Gaussian distribution can be
  approximated using multiple insert states to represent a single
  spacer region. The '--nspacer' option specifies the number of
  insert states used to represent each spacer. Note that this
  Gaussian approximation is only effective in conjunction with total
  probability training and scoring.
- --transpseudo <*value*> - Specify the value of the
  pseudocount used in converting transition counts to transition
  probabilities.
- --spacerpseudo <*value*> - Specify the value of the
  pseudocount used in converting transition counts to spacer
  self-loop probabilities.
- --description <*text*> - Specify descriptive text to
  be stored as a comment in the model file.
- --fim - A free-insertion module (FIM) is an insert state with
  1.0 probability of self-transition and 1.0 probability of exit
  transition. Thus, traversing such a state has zero transition cost.
  Specifying this option causes all spacers to be represented using
  FIMs.
- --keep-unused - By default, `mhmm` remove from the
  transition probability matrix all inter-motif transitions that are
  not observed in the data. This option allows those transitions to
  be retained. This option is only relevant if the model has a
  completely connected topology.
- --verbosity 1|2|3|4|5 - Set the verbosity level of the output
  to stderr. The default level is 2.
- --noheader - Do not put a header on the output file.
- --noparams - Do not list the parameters at the end of the
  output.
- --notime - Do not print the running time and host name at the
  end of the output.
- --quiet - Combine the previous three flags and set verbosity to
  1.

**Bugs:** None known.

Author: William
Stafford Noble.
